# An evidence map of clinical practice guideline recommendations and quality of non-pharmaceutical interventions for post-stroke emotional disorders

**DOI:** 10.3389/fneur.2025.1580799

**Published:** 2025-06-09

**Authors:** Ye Li, Jing Zhang, Jia-ji Li, Dan Zhao, Ling Tang, Ying-Hui Jin

**Affiliations:** ^1^Nursing Department of Dong fang Hospital, Beijing University of Chinese Medicine, Beijing, China; ^2^Department of Neurosurgery, Handan Central Hospital, Handan, China; ^3^Center for Evidence-Based and Translational Medicine, Zhong nan Hospital of Wuhan University, Wuhan, Hubei, China

**Keywords:** cerebral stroke, emotional disorder, non-pharmacological intervention, clinical practice guidelines, evidence-based medicine

## Abstract

**Background:**

Clinical practice guidelines (CPGs) have an indispensable role in guiding the selection of various non-pharmaceutical interventions (NPIs) for post-stroke emotional disorders (PSED). However, little is known about their quality and recommendations. This study aims to critically appraise the quality of existing NPIs for PSED CPGs and extract relevant recommendations, present the research distribution of various NPIs in an evidence map, and assist clinicians in making decisions.

**Methods:**

A systematic search was undertaken in PubMed, Embase, CINAHL, Web of Science, China National Knowledge Infrastructure, Wanfang, VIP, SinoMed, and international guideline developing institutions from origin to November 20, 2024, to identify the CPGs on NPIs for PSED. The CPGs finally selected were blindly evaluated by two reviewers using the Appraisal of Guidelines Research & Evaluation (AGREE) II instrument and the reporting quality was evaluated using the RIGHT statement. The overall agreement among reviewers was analyzed using intraclass correlation coefficient (ICC).

**Results:**

Nine guidelines were included and evaluated. Two CPGs were grade A (recommended) and seven CPGs were grade B (recommended with modification). The reporting rate of RIGHT ranged from 40.00 to 80.00%. Nine NPIs were extracted, and there were similarities and differences between the recommendations.

**Conclusion:**

This study provides specific direction for improving the quality of CPGs for NPIs for PSED, and provides useful information for clinicians and stakeholders, and provides a basis for clinical decision-making.

## 1 Introduction

Cerebral stroke is the second largest cause of death worldwide, accounting for 11.6% of the total number of deaths (1). It is characterized by a high incidence, high recurrence rate, high disability rate and high mortality rate, leading to an increased burden of disease around the world (1). Post-stroke emotional disorder (PSED) is one of the most common and serious complications, commonly occurring at all stages of the disease and its pathogenesis is still unclear (2). It includes post stroke depression (PSD), post-stroke anxiety (PSA), post-stroke comorbid anxiety and depression (PSCAD), post-stroke emotional imbalance (PSEI) and post-stroke anger proneness (PSAP) (3). Approximately one-third of stroke survivors develop some form of emotional disorder (2–4). Studies have shown (5, 6) that emotional disorders are closely related to patients’ prognosis. If patients are not treated in time, it will affect the recovery of neurological function and the ability to return to society, and even lead to increased mortality. There is no universally effective method for the treatment of PSED, and although drug therapy has a certain effect, there are many side effects (7). Some systematic reviews and meta-analyses have shown that non-pharmaceutical interventions (NPIs) can effectively reduce emotional symptoms and improve patient’s quality of life (7–9). Many authoritative organizations have issued a number of CPGs related to the treatment and rehabilitation of Stroke, which contain NPIs to help health care workers and patients to make local health care decisions (10).

The purpose of this study is to evaluate the quality of guidelines related to NPIs for PSED, to make relevant recommendations for NPIs use in PSED, to provide information for standardized practice and management, to identify potential directions that CPGs should focus on in the future, and to provide a reference for relevant policy development and clinical practice.

## 2 Materials and methods

### 2.1 Search strategy

We systematically searched the following databases: PubMed, Web of Science, Embase, CINAHL, China National Knowledge Infrastructure (CNKI), Wanfang, VIP, SinoMed, YiMaiTong. We also hand-searched 6 databases of international guideline developing institutions: Guideline International Network (GIN), Registered Nurses’ Association of Ontario (RNAO), Scottish Intercollegate Guidelines Network (SIGN), National Institute for Health and Care Excellence (NICE), National Guideline Clearinghouse (NGC), and New Zealand Guidelines Group (NZGG). Articles were retrieved by combining subject terms and free terms, from origin to November 20, 2024. The full search strategies are shown in Supplementary material 1.

### 2.2 Study selection

In our study, the inclusion criteria were: guidelines which provided recommendations regarding NPIs for PSED and included access to the full text. Both evidence-based clinical practice guidelines and consensus-based clinical practice guidelines (EB-CPGs and CB-CPGs) were included, and the guidelines had to at least contain details of evidence retrieval and literature evaluation. We have described both consensus statements and expert opinions as CB-CPGs (11). The CPGs had to include NPIs for PSED. The most commonly included NPIs were: psychotherapy, social support therapy, traditional Chinese medicine non-pharmacological therapies, and physical therapy (12). If guidelines were available in multiple languages (such as English and Chinese), only the version in the original language was eligible for inclusion. In cases of updated guidelines, only the most recent version was considered. Exclusion criteria were editorial or correspondence articles that summarized organizational clinical practice guidelines.

### 2.3 Data extraction

Two reviewers (LY and ZJ) screened all references independently, and any ambiguities were resolved by a third reviewer (LJJ). The two reviewers developed a purpose-designed data extraction sheet covering the basic characteristics, which included the following: title, guideline development institution, publication year, development country, guideline type, journal, target population, number of references and funding support. This was used to record extracted NPIs for PSED both where there was a clear level of evidence or recommendation and also where suggested or conclusive intervention methods were concealed within the paragraphs of guidelines written in flowing text format.

### 2.4 Guideline quality assessment

The methodological quality of the guidelines was evaluated by two experienced reviewers (LY and ZJ) using the Application of Guidelines for Research and Evaluation (second version) (AGREE II) (10), which consists of 23 items organized into the six domains. In case of disagreement, the decision was made after discussion with a third reviewer (ZD). The standardized scores for each of the individual AGREE domains were calculated: a higher score indicates a higher quality of the domain.^1^ According to the standardized score of each field in the guide, the recommendations were divided into three levels: ≥60% of 6 domains were rated as Grade A (recommended); more than three domains with≥30%, but less than< 60% domains were rated as Grade B (recommended with modifications); more than three domains with <30% were rated as Grade C (not recommended) (13–15).

Two reviewers (LY and ZJ) were trained to independently assess the reporting quality appraisals of CPGs using the Reporting Items for practice Guidelines in Healthcare (RIGHT) checklist (16), which consists of 35 sub-items grouped into seven domains and the criteria for assessing the items are “Yes” (guideline reported majority information), “No” (relevant information on the item was not reported), and “NA” (not applicable, the item did not need to be evaluated due to certain characteristics of the guideline). Any inconsistency was resolved by a third reviewer (ZD) after a thorough discussion by the two reviewers. We calculated the domain reporting rate for each included guideline, and calculated the mean reporting rate of each domain for all included guidelines (17–19).

### 2.5 Data synthesis and analysis

The reviewers thoroughly read, carefully understood, and accurately interpreted the implications of the recommendations regarding NPIs for PSED, and then classified them into different themes based on their characteristics. Both the evidence quality and the recommendation strength of the same NPIs were compared and analyzed. We used visual clinical pathways to display NPIs related to different emotional states and utilized Intraclass Correlation Coefficients (ICCs) to evaluate the level of consistency and reliability between the two researchers (20). The degree of agreement could be classified into poor (<0.40), moderate (0.40–0.75), good (≥0.75) (21, 22). Data were entered using an excel form and data analysis was performed using the “SPSSAU Data Analysis Platform.”^2^ A bubble plot was designed to comprehensively display the overall quality of each eligible CPG, the X-axis denoted the average reporting rate of the RIGHT checklist, and the Y-axis listed the AGREE II total score. An evidence map was used to summarize the strength distribution of the guideline recommendations.

## 3 Results

### 3.1 Search results

**Figure 1:**
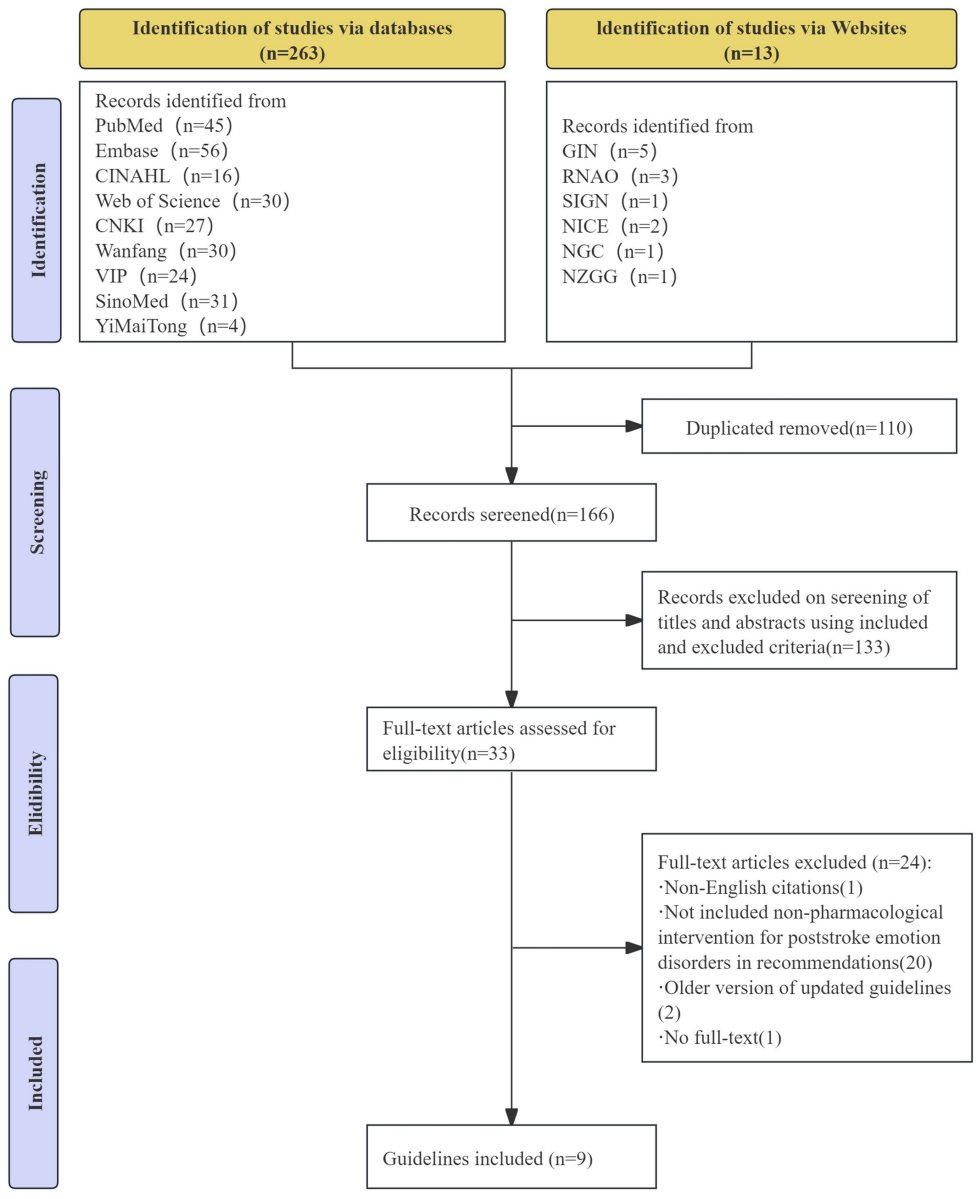
Flow diagram of literature screening.

A total of 276 records were retrieved through the literature search. There were 166 duplicates, and 33 irrelevant records: nine CPGs (23–31) proved eligible (Figure 1). Five of these were EB-CPGs (23, 24, 27, 28, 30) and four were CB-CPGs (25, 26, 29, 31). Table 1 presents the characteristics of the eligible CPGs.

**Table 1:** Characteristics of eligible guidelines.

Developer and Guideline, year	Journal	Country	Guideline type	Target user	Number of References	Funding source
UWHMC, 2012 (26)	Journal of general internal medicine	United States	EB-CPGs	Healthcare providers	67	None
AHA/ASA, 2017 (27)	Stroke	United States	EB-CPGs	Healthcare providers	102	None
CSPM, 2018 (28)	Brazilian Journal of Psychiatry	China	CB-CPGs	Stroke clinicians and related workers	126	Jiangsu Provincial Special Program of Medical Science
CGHCMRA, 2019 (29)	Chinese Journal of Geriatric Care	China	CB-CPGs	Rehabilitation physician	43	None
CSC, 2020 (30)	International Journal of Stroke	Canada	EB-CPGs	Healthcare professionals and system leaders	106	Heart and Stroke Foundation, Canada
CSA, 2020 (31)	Stroke and Vascular Neurology	China	EB-CPGs	Healthcare professionals	46	Chinese Stroke Association Guidelines Writing Committee
HMHA, 2020 (34)	Journal of Clinical Psychosomatic Diseases	China	CB-CPGs	Stroke clinicians	34	None
CBNIRC, 2022 (33)	Indian Journal of Psychiatry	India	EB-CPGs	Psychiatry	110	None
BCPA, 2023 (36)	Chinese Journal of Stroke	China	CB-CPGs	Stroke clinicians	6	National Key Research and Development Program of China

UWHMC, University of Washington Harborview Medical Center; AHA/ASA, Heart Association/American Stroke Association; CSPM, Chinese Society of Psychosomatic Medicine; CGHCMRA, Chinese Geriatric Health Care and Medical Research Association; CSC, Canadian Stroke Consortium; CSA, Chinese Stroke. Association; CHMHA, Chinese Henan Mental Health Association; CBNIRC, Clinical Brain Neuropsychiatric Institute and Research Center; BCPA, Beijing Cerebrovascular disease Prevention Association.

### 3.2 Quality of CPGs

The overall quality of the included guidelines was acceptable, two of which were rated as grade A, and seven were rated as grade B. The results of the AGREE II score are shown in Table 2. The details of the AGREE II scores are reported in Supplementary material 2. Since there were no uniform criteria according to AGREE II instrument and other previous studies had defined a uniform criterion for overall quality (23, 32). We defined domains with scores <50% as lower scores, 50–70% as moderate, and >70% as high scores. Among the six domains of AGREE II, Domain 1 (Scope and Purpose), Domain four (Clarity of Presentation) and Domain six (Editorial Independence) scores were relatively high. Scores were moderate in Domain two (Stakeholder Involvement), 44.44% of CPGs scores <60%, mainly due to “The views and preferences of the target population (patients, public, etc.) have been sought” had low scores, indicating that these CPGs did not adequately take into account the values and preferences of the patients in their development. Domain five (Applicability) domain scores have been scored relatively low, because the CPGs did not adequately describe the advantages and disadvantages in the application process or specify implementation strategies, etc. Domain three (Rigor of Development) had the lowest score compared with other domains. In this domain, 66.67% of the guidelines scored < 60%. This was mainly due to poor rigor in the formulation of the guidelines. There was a lack of detailed description of the methods for forming recommendations, the strength of recommendations, the level of supporting evidence, the external review process before publication of the guidelines, and the steps for updating the guidelines. The ICC statistics for the assessment between the two reviewers regarding the AGREE II domains varied from 0.61 to 0.98 (*p* < 0.001), reflecting that inter-rater reliability was good to excellent.

**Table 2:** Standardized scores of guidelines as measured by AGREE II instrument.

Guideline, year	Scope and purpose	Stakeholder involvement	Rigor of development	Clarity of presentation	Applicability	Editorial independence	The number of domains with standardized scores ≥ 60%	ICC	*p*	Strength of recommendation
UWHMC, 2012 (26)	88.89	47.22	43.75	88.89	22.92	87.50	3	0.79	<0.001	B
AHA/ASA, 2017 (27)	97.22	61.11	65.63	88.89	52.08	87.50	5	0.83	<0.001	B
CSPM, 2018 (28)	91.67	41.67	19.79	80.56	33.33	91.67	3	0.52	<0.001	B
CGHCMRA, 2019 (29)	91.67	66.67	29.17	88.89	50.00	87.50	4	0.77	<0.001	B
CSC, 2020 (30)	100.00	72.22	97.92	100.00	81.25	95.83	6	0.98	<0.001	A
CSA, 2020 (31)	91.67	77.78	66.67	91.67	60.41	91.67	6	0.97	<0.001	A
HMHA, 2020 (34)	91.67	52.78	9.38	91.67	37.50	79.17	3	0.79	<0.001	B
CBNIRC, 2022 (33)	100.00	52.78	38.54	80.56	43.75	87.50	3	0.79	<0.001	B
BCPA, 2023 (36)	86.11	77.78	20.83	91.67	41.67	79.17	4	0.61	<0.001	B
Mean ± SD	93.21 ± 4.83	61.11 ± 13.32	43.52 ± 28.41	89.20 ± 5.96	46.99 ± 16.90	87.50 ± 5.51	-	-	-	-

**Figure 2:**
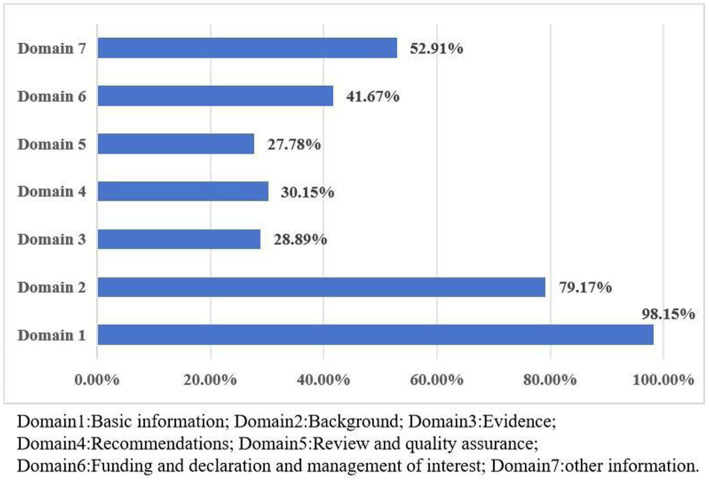
Reporting of RIGHT items in CPGs and Supplementary material 3.

**Figure 3:**
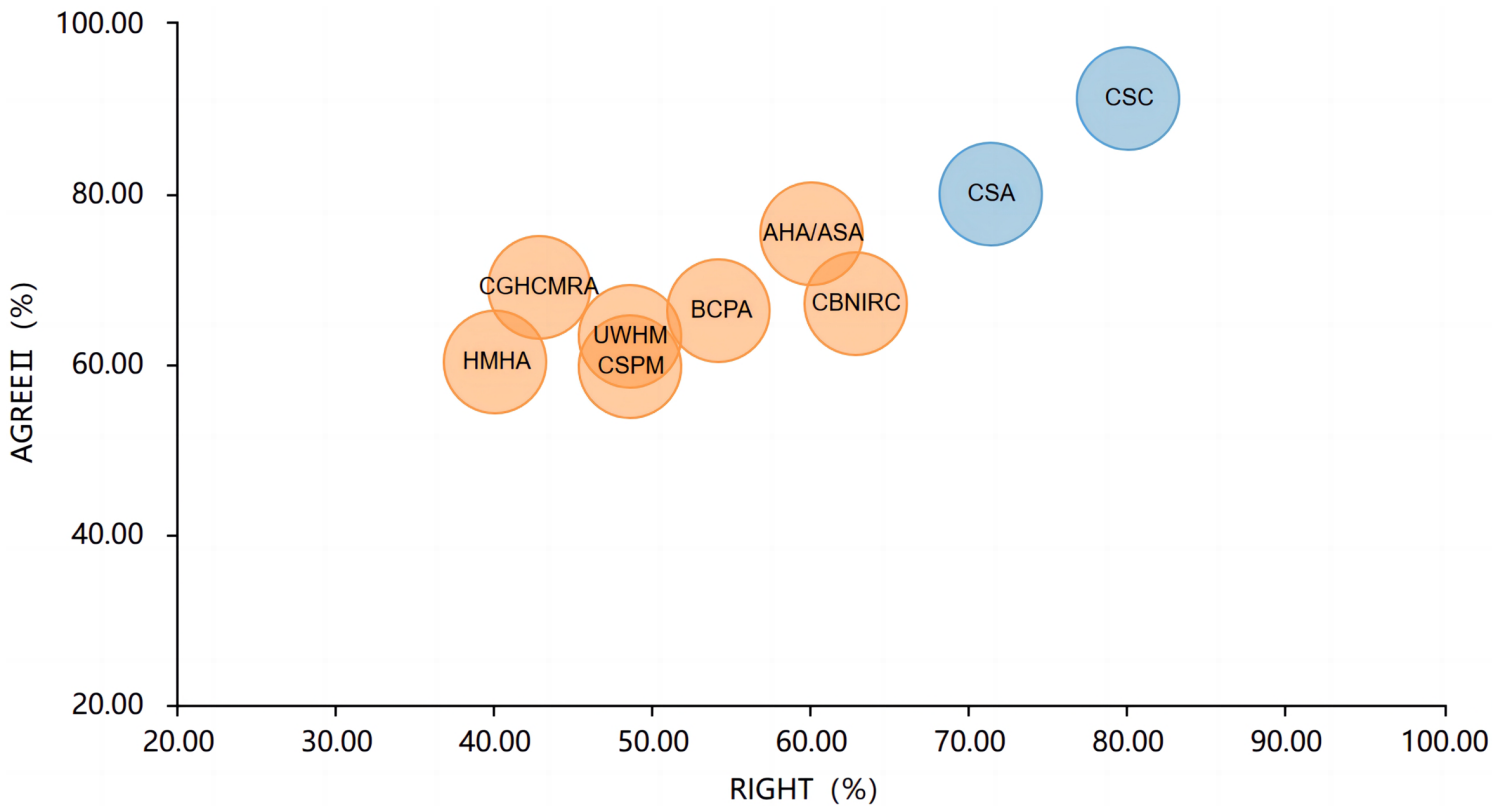
Grading and analysis of reporting and methodological quality of CPGs.

The overall RIGHT reporting rate of the nine guidelines ranged from 40.00% to 80.00%, with an average reporting rate of 56.51%. Among the seven domains, the “basic information” domain had the highest reporting rate (98.15%), followed by “background” domain (79.17%), and the “Review and Quality Assurance” domain got the lowest reporting rate (27.78%). Items with serial reporting defects included item 10b (Indicate how the outputs were selected and sorted), item14a (values and preferences of the target population), item14b (other cost and resource applications), item14c (other factors taken into consolidation), item16 (while the draft guideline undertook independent review), item17 (while the guideline was subject to a quality assurance process). The report quality assessment for all domains is shown in Figure 2. A bubble plot showed the overall quality of each guideline (Figure 3), the two blue spheres represented relatively high-quality CPGs, namely CSC (27) and CSA (28), and the seven CPGs with yellow spheres were relatively low-quality.

### 3.3 Recommendations on NPIs for PSED

**Figure 4:**
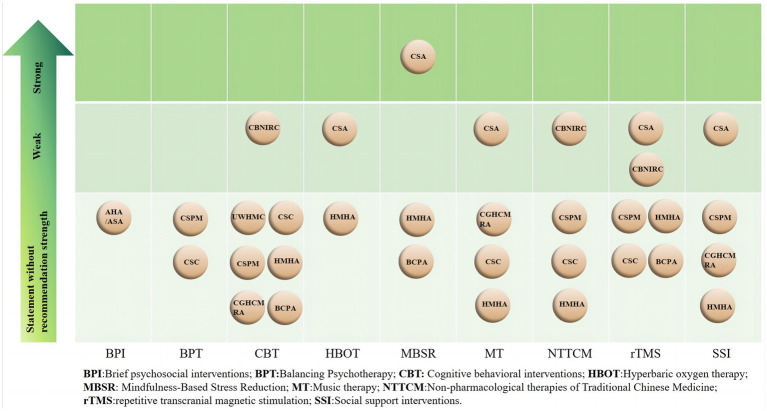
Evidence map of CPG recommendations on NPIs for PSED.

**Figure 5:**
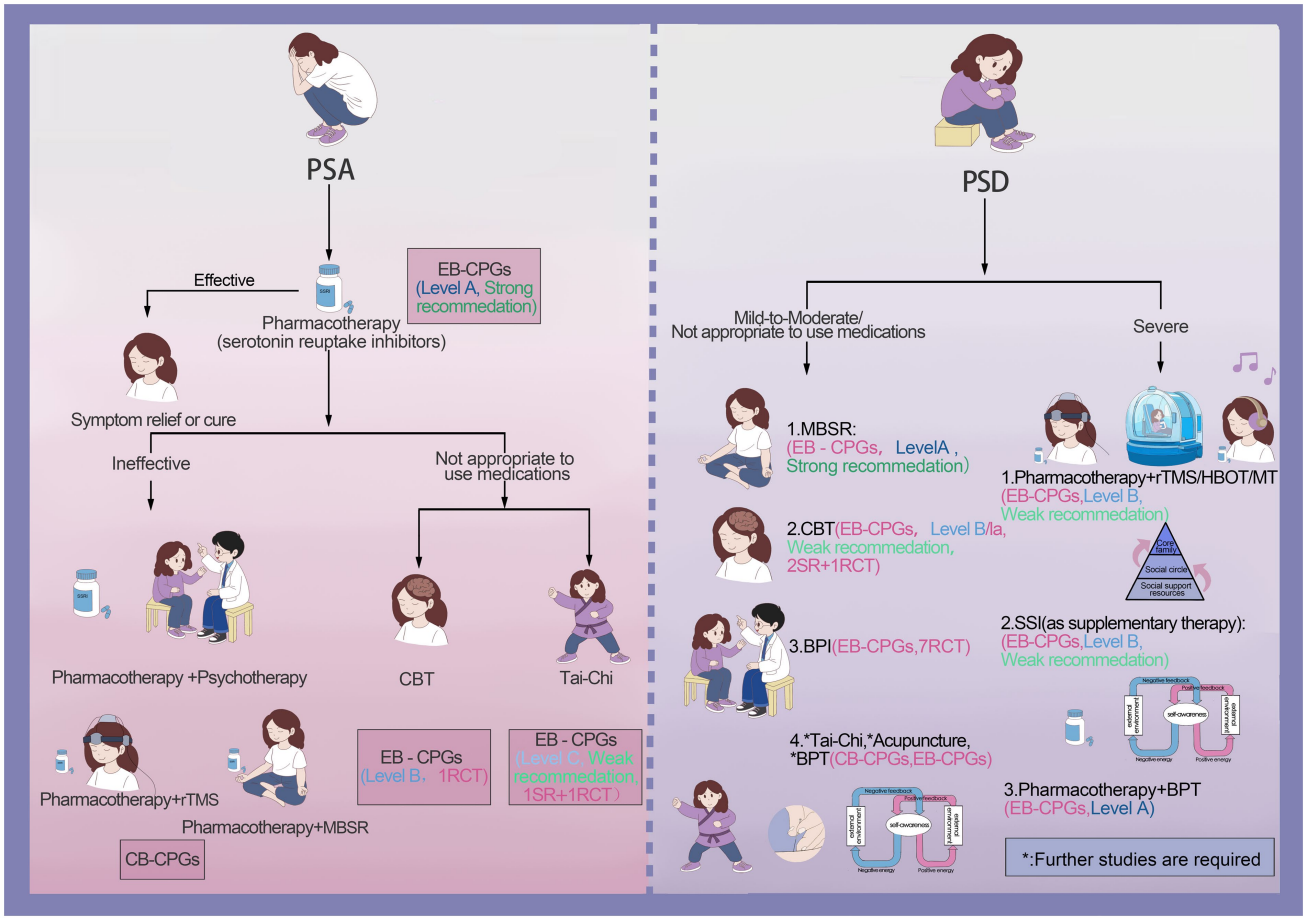
Clinical pathway map of NPIs.

We summarized nine NPIs from the eligible CPGs (23–31), and only four CPGs (23, 27, 28, 30) identified the level of evidence and/or strength of recommendation. Details regarding the level of evidence and the strength of the recommendations in the different grading systems are described in Supplementary material 4, and the evidence quality and recommendation strength are reported in Supplementary material 5. The main types of PSED are PSD and PSA in the eligible CPGs. There were similarities and differences in recommendations for NPIs, and differences in recommendation strength (Figure 4). The visual clinical pathways of NPIs for PSD and PSA are shown in Figure 5.

With regard to the use of psychotherapy for PSD, three CPGs (28, 29, 31) recommended using mindfulness-based stress reduction (MBSR) to improve symptoms, one EB-CPG (28) gave this a strong recommendation, while two CB-CPGs gave no recommendation strength. Seven CPGs (23, 25–27, 29–31) recommended cognitive behavioral interventions (CBT) to improve symptoms of PSD, but only one EB-CPG (30) recommended CBT to improve mild PSD and this gave a weak recommendation. There were four CPGs (26–29) that recommended that music therapy (MT) in combination with drug therapy was more effective for severe PSD, and only one guideline (28) gave this a weak recommendation. Balancing Psychotherapy (BPT) integrated treatment modalities from diverse psychological schools to help eliminate the cause of PSD. Specifically, two CPGs (25, 27) had put forward the suggestion that BPT could be utilized as an auxiliary treatment modality for PSD. However, they failed to offer precise recommendations. The guidelines suggested that psychotherapy can be the primary treatment option for patients suffering from mild to moderate PSD, and also for those who had poor medication compliance or experienced adverse drug reactions (25, 29). However, for patients with severe PSD or those with complications, it was recommended that psychotherapy be used as an adjunctive treatment to pharmacotherapy (27, 29).

In terms of social intervention treatment, social support interventions (SSI) were recommended as a supplementary treatment for PSD (25, 26, 28, 29). It was recommended that family members, friends, and colleagues encourage patients to participate in social activities, and one EB-CPG (28) gave this a weak recommendation. Additionally, another EB-CPG (24) recommended the utilization of brief psychosocial interventions (BPI). The supporting evidence was derived from seven RCTs (*n* = 775) and showed the effectiveness of BPI. However, since the designs of these studies were not rigorous, it was uncertain whether antidepressants played an auxiliary role, so no specific recommendation strength was provided.

Some NPIs suggested that traditional Chinese medicine had potential effects in treating PSD. Two CB-CPGs (25, 29) recommended the application of acupuncture and Tai-Chi to alleviate the symptoms of PSD. Another EB-CPG (27) pointed out that compared with drug therapy, acupuncture was regarded as effective in the treatment of PSD. Nevertheless, acupuncture has not been extensively used in the healthcare systems of different countries. Its clinical effectiveness needs further study. Hence, no recommendation strength was given.

Another type of NPI for PSD was physical therapy. A weak recommendation was given for the combined application of repetitive transcranial magnetic stimulation (rTMS) and drugs in the treatment of severe PSD (31, 33). Hyperbaric oxygen therapy (HBOT) has also been shown to improve the metabolism of brain tissue and promote brain function recovery (31, 34). One EB-CPG (31) recommended using HBOT combined with drug treatment for severe PSD and gave it a weak recommendation.

For the NPIs for PSA, the CPGs recommended drug therapy as the first choice (23, 27, 28, 31). One CB-CPG (31) recommended that when the efficacy of drug therapy was not satisfactory, a combination approach could be adopted. Specifically, drug therapy could be paired with psychotherapy such as CBT, or joined with rTMS, or integrated with MBSR, all with the aim of enhancing the therapeutic outcome. When patients were unable to take medication, Tai-Chi was recommended for the treatment of PSA, and was given a weak recommendation (30), nevertheless, further validation of the effect is necessary. In addition, one CPG (27) recommended CBT for the treatment of PSA. However, the evidence was derived from one RCT and the strength of recommendation was not provided.

## 4 Discussion

NPIs possess the advantages of high safety, a broad applicable population, as well as the enhancement of patients’ self-management capabilities (34). The majority of stroke patients were the elderly, who are likely to have multiple underlying co-morbidities, such as diabetes, hypertension and so on (35). Typically, these patients need to take a long term drug therapy to control their conditions, with resultant liver and kidney damage (33, 36). The use of NPIs could reduce the side effects of drugs. They focus on the cultivation of patients’ psychological adjustment ability and a positive attitude toward life, thereby facilitating the overall rehabilitation process (23, 32).

The overall quality of the nine CPGs in this study still needs improvement. Stakeholder Involvement, Rigor of Development and Applicability were underrepresented in all CPGs. All the guidelines had elaborated on the target populations (23–31). However, the viewpoints and preferences of these people (patients, the public, etc.) had not been taken into account. Future guideline developers could adopt methods such as questionnaires and interviews (35, 37) to understand the viewpoints and choices of the target populations, consider their actual feelings and needs, provide treatment options that meet their expectations, improve treatment compliance, and at the same time reflect the humanization of medical care.

In the rigor of the development, most of the guidelines did not describe in detail the strength of the recommendations and the level of evidence. The methods for formulating the recommendations (23–26, 29–31), the review of CPGs by external experts, their publication (23, 25, 26, 28–31), and the procedures for updating the CPGs were also inadequately reported (23–26, 29–31). The applicability score was the lowest among all CPGs. Only two CPGs considered the facilitator and barrier factors in the application process (24, 27), and six CPGs provided suggestions for applying the recommendations to practice, but lacked detailed implementation strategies (23–27, 29), Similarly, only two CPGs stated the potential resource impact of applying these recommendations (27, 28), but did not report the potential costs. Thus, the deficiency in the clinical applicability of the guidelines implied that, throughout the development process, insufficient attention was paid to transforming the recommendations into clinical applications. The assessment results of the RIGHT list indicated that the domain with the lowest score was Review and Quality Assurance. Similar to AGREE II, there were also deficiencies in such aspects as “the views and preferences of the target population,” “cost and resource impact,” “independent review” and “quality assurance.” Consequently, the future development of CPGs covering NPIs for PSED needs to center on the aspects of those low-scoring items, with the aim of enhancing the overall guideline quality.

The latest systematic reviews and a meta-analysis indicate that NPIs are effective in improving mood and quality of life for stroke survivors (12, 38). So far, the CPGs relating to NPIs for PSED only cover the two different types of emotional disorders: namely PSD and PSA, with PSD being the predominant focus. The recommendations of the guidelines are inconsistent and most of the guidelines did not give a clear recommendation (23–27, 29, 31), while some provided merely the level of evidence (23, 27, 28, 30). This indicates a lack of uniformity in the evidence related to NPIs for PSD and PSA, as well as varying degrees of research depth in this area.

Our research found that the NPIs strongly recommended by the guideline were MBSR (Level A) which (28) was developed by a multidisciplinary team in China, and the patient group it targeted was Chinese adult stroke patients. However, since the guideline was not specifically designed for PSD, it did not mention the specific implementation methods of MBSR and so lacks clinical applicability. Relevant studies have suggested that MBSR can be one-time or multi-stage, using techniques such as diaphragmatic breathing, mind–body scan and mindful imaging (39). The subtypes of the intervention include mindful cognitive therapy, mindful stress reduction therapy and mindful self-care (40). Nevertheless, there are several obstacles to the clinical application of MBSR. For instance, there is a scarcity of professionals well-versed in both the theory and practice of MBSR. Additionally, patients’ cultural backgrounds and religious beliefs could pose challenges, which might potentially explain why other CPGs did not provide relevant recommendations.

The CPGs made weak recommendations for NPIs including Tai Chi and CBT. The evidence was derived from systematic reviews and RCTs (26, 30, 33, 36). SSI as an adjuvant therapy has also been given a weak recommendation (Level B), and the evidence level for the combination of drugs with rTMS, HBOT or MT was Level B. Due to the lack of high-quality evidence in the studies of these NPIs, CPGs made weak recommendations, which have also been verified by other research (38, 41). In addition, since Tai Chi research was influenced by Chinese data, the promotion and application of Tai Chi in other countries or regions may be restricted. Therefore, when using Tai Chi as an intervention, its localization should be fully considered, and the quality of original research should be improved. A recent systematic review and meta-analysis have shown that acupuncture was the most effective non-pharmacological intervention for improving patients’ emotions (38). In addition, acupuncture had a positive impact on other stroke-related symptoms, such as physical function, speech, and cognition (42, 43), further enhancing the overall treatment efficacy for improving emotions. This was consistent with the viewpoints expressed in the CPGs included in our study (25, 27, 29). However, due to acupuncture’s extensive theoretical system, complex mechanism of action, and high operational requirements, it had not been widely applied globally and further in-depth research was still needed.

Our results indicate that the CPGs included in this study only recommend different NPIs, but detailed clinical implementation plans for NPIs are still lacking. Most of the existing CPGs were focused on PSD. In contrast, other CPGs incorporate the treatment and management of PSED as a segment of their content. However, the recommendations regarding PSED tend to be neither sufficiently clear nor comprehensive. Stroke patients frequently suffer from multiple emotional problems (44). However, there were few studies on emotional states such as PSA, PSCAD, PSEI, PSAP, and extremely few studies on the comorbidity of different emotional states (45). Future research should explore the efficacy of NPIs for different emotional states. By integrating the preferences and demands of patients as well as other stakeholders, and utilizing a variety of tools and resources, personalized intervention programs need to be formulated to further enhance the evidence quality of NPIs for PSED. Subsequently, this will provide high-quality evidence support for the formulation of NPIs guidelines for PSED, and facilitate clinicians making more scientific and feasible clinical decisions.

To our knowledge, this is the first comprehensive systematic study on the existing CPGs for NPIs for PSED. We have used the most common and comprehensive assessment tools, namely AGREE II and RIGHT, to evaluate the methodological quality and reporting quality of the CPGs. We have comprehensively extracted the recommendations, including the recommended content hidden in paragraph style texts. Additionally, we have also used an evidence map to clearly display the strength distribution of the recommendations, and visualized the clinical intervention pathways in different emotional states to assist in clinical decision-making. However, there are several limitations in our study. First, although we used systematic and standardized methods for retrieval, only English and Chinese CPGs were eventually included, and this might result in potential biases. Only two reviewers evaluated the included guidelines, but the consistency between the reviewers was relatively good.

## 5 Conclusion

The overall quality of the included guidelines still needs to be enhanced, especially in aspects such as stakeholder involvement, rigor of development and clinical applicability. The existing CPGs have provided recommendations on various NPIs for the treatment of PSD and PSA. When applying them to clinical practice, comprehensive considerations should be made by integrating patients’ wishes and the medical environmental factors. In the future, when formulating guidelines for NPIs for PSED, the differences in the emotional stages of patients, the clinical applicability of the guidelines, as well as potential resource and implementation barriers should be taken into account, and timely updates and specific recommendations should be provided. Our research findings can help clinicians choose the most suitable guidelines and recommendations based on the actual situation and accessible resources, promote the standardized application of NPIs in patients with PSED, and improve the quality of the development of clinical practice guidelines for NPIs for PSED. At the same time, they also provide a reference for future guideline formulation and research.

## Footnotes

^1^ https://www.agreetrust.org

^2^ https://spssau.com/index.html
