# Supplementary material for: An evidence map of clinical practice guideline recommendations and quality of non-pharmaceutical interventions for post-stroke emotional disorders
Source: Front Neurol. 2025 Jun 9;16:1580799. doi: 10.3389/fneur.2025.1580799 (PMC12183077; doi:10.3389/fneur.2025.1580799)
Supplement: Supplementary file 1 [file Table_1.docx]

**S Table 1. Search** **strategy**

| **Databases [Platform]**  *Search from origin to November 20, 2024.* | **Query** |
| --- | --- |
| **PubMed** | ("stroke"[MeSH Terms] OR "stroke"[Title/Abstract] OR "cerebrovascular accident"[Title/Abstract] OR "cerebral vascular accident"[Title/Abstract] OR "cerebrovascular disorder"[Title/Abstract] OR "cerebral vascular disorders"[Title/Abstract] OR "cerebral infarction"[Title/Abstract] OR "brain ischemia"[Title/Abstract]) OR "cerebral hemorrhage"[Title/Abstract]) AND ("post-stroke depression"[Title/Abstract] OR "post-stroke anxiety"[Title/Abstract] OR "post-stroke comorbid anxiety and depression"[Title/Abstract] OR "post-stroke emotional imbalance"[Title/Abstract] OR "post-stroke anger proneness"[Title/Abstract] OR "emotional disorders"[Title/Abstract]) AND ("Guidelines as Topic"[MeSH Terms] OR "guideline*"[Title] OR "clinical practice guidelines"[Title] OR "CPG*"[Title] OR "practice guideline*"[Title] OR "recommendation*"[Title] OR "good practice statement*"[Title] OR "consensus"[MeSH Terms] OR "consensus"[Title] OR "expert consensus"[Title]) |
| **Web of Science** | (TS=(stroke) OR TS=(cerebrovascular accident) OR TS=(cerebral vascular accident) OR TS=(cerebrovascular disorder) OR TS=(cerebral vascular disorders) OR TS=(cerebral infarction) OR TS=(brain ischemia) OR TS=(cerebral hemorrhage)) AND (TS=(post-stroke depression) OR TS=(post-stroke anxiety) OR TS=(post-stroke comorbid anxiety and depression) OR TS=(post-stroke emotional imbalance) OR TS=(post-stroke anger proneness) OR TS=(emotional disorders) OR TS=( depress) OR TS=(depressive disorder)) AND (TI=(guideline*) OR TI=(clinical practice guidelines) OR TI=(CPG*) OR TI=(consensus) OR TI=(practice guideline) OR TI=(recommendation*) OR TI=(consensus) OR TI=(expert consensus)) |
| **Embase** | ('cerebrovascular accident'/exp OR ('cerebrovascular accident':ab,ti OR 'cerebral vascular accident':ab,ti OR 'cerebrovascular disorder':ab,ti OR 'cerebral vascular disorders':ab,ti OR 'cerebral infarction':ab,ti OR 'brain ischemia':ab,ti OR 'cerebral hemorrhage':ab,ti)) AND ('post-stroke depression':ab,ti OR 'post-stroke anxiety':ab,ti OR 'post-stroke comorbid anxiety and depression':ab,ti OR 'post-stroke emotional imbalance':ab,ti OR 'post-stroke anger proneness':ab,ti OR 'emotional disorders':ab,ti)) AND ('practice guideline'/exp OR 'practice guideline':ti OR 'clinical practice guideline':ti OR cpgs:ti OR 'recommendations':ti OR 'good practice statement*':ti OR 'consensus'/exp OR 'consensus':ti OR 'expert consensus':ti) |
| **CINAHL** | (MM ("stroke") OR SU ("stroke" or "cerebrovascular accident" or "cerebral vascular accident" or "cerebrovascular disorders" or "cerebral vascular disorders" or "cerebral infarction" or "brain ischemia" or "cerebral hemorrhage") AND (SU ("post-stroke depression" or "post-stroke anxiety" or "post-stroke comorbid anxiety and depression" or "post-stroke emotional imbalance" or "post-stroke anger proneness"or "post-stroke emotional imbalance") AND (MM ( Consensus or "Practice Guidelines" ) OR TI ( guideline* or "clinical practice guidelines" or "CPGs" or "practice guideline*" or recommendation* or "good practice statement*" or consensus or "expert consensus")) |
| **China National Knowledge Infrastructure** | (TKA= '卒中' OR TKA= '脑卒中' OR TKA= '中风' OR TKA= '脑血管意外' OR TK = '脑出血' OR TKA= '脑梗死' OR TKA= '脑血管事件' OR TKA= '脑缺血') AND (TKA= '卒中后抑郁' OR TKA= '中风后抑郁' OR TKA= '卒中后焦虑' OR TKA= '中风后焦虑' OR TKA = '焦虑抑郁共病' OR TKA = '情绪失衡' OR TKA = '愤怒倾向' OR TKA = '情绪障碍') AND (TI= '共识' OR TI= '指南' OR TI= '临床实践指南' TI= '推荐意见') |
| **WanFang** | 主题:(卒中 or 脑卒中 or 中风 or 脑血管意外 or 脑出血 or 脑梗死 or 脑血管事件 or 脑缺血 ) and 主题:(卒中后抑郁 or 中风后抑郁 or 卒中后焦虑 or 中风后焦虑 or 焦虑抑郁共病 or 情绪失衡 or 愤怒倾向 or 情绪障碍) and 题名:(指南 or临床实践指南or 共识 or 推荐意见) |
| **VIP** | M=(卒中 OR 脑卒中 OR 中风 OR 脑血管意外 OR 脑出血 OR 脑梗死 OR 脑血管事件 OR 脑缺血) AND M=(卒中后抑郁 OR 中风后抑郁 OR 卒中后焦虑 OR 中风后焦虑 OR 焦虑抑郁共病 OR 情绪失衡 OR 愤怒倾向 OR 情绪障碍 ) AND ( T=指南 OR 临床实践指南OR共识 OR推荐意见) |
| **SinoMed** | ("愤怒倾向"[常用字段] OR (("抑郁"[常用字段] OR "Depression"[常用字段] OR "抑郁症状"[常用字段] OR "情绪抑郁"[常用字段] OR "抑郁"[主题词]) OR "卒中后抑郁"[常用字段] OR "中风后抑郁"[常用字段] OR ("焦虑"[常用字段] OR "Anxiety"[常用字段] OR "紧张"[常用字段] OR "过度警觉"[常用字段] OR "焦虑"[主题词]) OR "卒中后焦虑"[常用字段] OR "中风后焦虑"[常用字段] OR "焦虑抑郁共病"[常用字段] OR "情绪失衡"[常用字段] AND ("情绪障碍"[常用字段] OR "Mood Disorders"[常用字段] OR "情感障碍"[常用字段] OR "情绪障碍"[主题词]))) AND (("卒中"[常用字段] OR "Stroke"[常用字段] OR "脑血管意外"[常用字段] OR "脑中风"[常用字段] OR "CVA(脑血管意外)"[常用字段] OR "CVAs(脑血管意外)"[常用字段] OR "脑血管中风"[常用字段] OR "脑卒中"[常用字段] OR "急性中风"[常用字段] OR "急性卒中"[常用字段] OR "急性脑血管意外"[常用字段] OR "急性脑卒中"[常用字段] OR "卒中"[主题词]) OR ("脑卒中"[常用字段] OR "卒中"[常用字段] OR "Stroke"[常用字段] OR "脑血管意外"[常用字段] OR "脑中风"[常用字段] OR "CVA(脑血管意外)"[常用字段] OR "CVAs(脑血管意外)"[常用字段] OR "脑血管中风"[常用字段] OR "急性中风"[常用字段] OR "急性卒中"[常用字段] OR "急性脑血管意外"[常用字段] OR "急性脑卒中"[常用字段] OR "卒中"[主题词]) OR ("中风"[常用字段] OR "Stroke"[常用字段] OR "卒中"[常用字段] OR "风痱"[常用字段] OR "中风"[主题词]) OR ("脑血管意外"[常用字段] OR "卒中"[常用字段] OR "Stroke"[常用字段] OR "脑中风"[常用字段] OR "CVA(脑血管意外)"[常用字段] OR "CVAs(脑血管意外)"[常用字段] OR "脑血管中风"[常用字段] OR "脑卒中"[常用字段] OR "急性中风"[常用字段] OR "急性卒中"[常用字段] OR "急性脑血管意外"[常用字段] OR "急性脑卒中"[常用字段] OR "卒中"[主题词]) OR ("脑出血"[常用字段] OR "Cerebral Hemorrhage"[常用字段] OR "大脑脑出血"[常用字段] OR "大脑出血"[常用字段] OR "脑实质出血"[常用字段] OR "脑内出血"[常用字段] OR "脑出血"[主题词]) OR ("脑梗死"[常用字段] OR "Brain Infarction"[常用字段] OR "脑静脉梗塞"[常用字段] OR "前脑循环梗死"[常用字段] OR "后循环脑梗塞"[常用字段] OR "脑梗塞"[常用字段] OR "前循环脑梗塞"[常用字段] OR "脑梗死"[主题词]) OR "脑血管事件"[常用字段] OR ("脑缺血"[常用字段] OR "Brain Ischemia"[常用字段] OR "缺血性脑病"[常用字段] OR "脑缺血症"[常用字段] OR "脑缺血"[主题词])) AND (("指南"[标题] OR "临床实践指南"[标题] OR "共识"[标题] OR "推荐意见"[标题] ) |
| **Guideline websites**   - Guidelines International Network (GIN, <https://g-i-n.net/)> - National Institute for Health and Clinical Excellence (NICE, <https://www.nice.org.uk/)> - Scottish Intercollegiate Guidelines Network (SIGN, https://www.sign.ac.uk/) - Registered Nurses Association of Ontario(RNAO,https://rnao.ca/) - New Zealand Guidelines Group(NZGG,http://www.nzgg.org.nz/) | post-stroke depression OR post-stroke anxiety OR post-stroke comorbid anxiety and depression OR post-stroke emotional imbalance OR post-stroke anger proneness OR emotional disorders |
| **Other websites (****Yimaitong)** | 卒中 or 脑卒中 or 中风 or 脑血管意外 or 脑出血 or 脑梗死 or 脑血管事件 or 脑缺血 |
