# Supplementary material for: An evidence map of clinical practice guideline recommendations and quality of non-pharmaceutical interventions for post-stroke emotional disorders
Source: Front Neurol. 2025 Jun 9;16:1580799. doi: 10.3389/fneur.2025.1580799 (PMC12183077; doi:10.3389/fneur.2025.1580799)
Supplement: Supplementary file 2 [file Table_2.docx]

**S Table 2. The original scores given by the two appraisers for AGREE Ⅱ**

|  | **UWHMC,**  **2012** | | **AHA/ASA,**  **2017** | | **CSPM,**  **2018** | | **CGHCMRA,**  **2019** | | **CSC,**  **2020** | | **CSA,**  **2020** | | **HMHA,**  **2020** | | **CBNIRC,**  **2022** | | **BCPA,**  **2023** | |
| --- | --- | --- | --- | --- | --- | --- | --- | --- | --- | --- | --- | --- | --- | --- | --- | --- | --- | --- |
|  | A1 | A2 | A1 | A2 | A1 | A2 | A1 | A2 | A1 | A2 | A1 | A2 | A1 | A2 | A1 | A2 | A1 | A2 |
| **Domain 1:Scope and Purpose** | 20 | 18 | 21 | 20 | 21 | 18 | 21 | 18 | 21 | 21 | 21 | 18 | 21 | 18 | 21 | 21 | 19 | 18 |
| *The scaled domain score* | **88.89%** | | **97.22%** | | **91.67%** | | **91.67%** | | **100%** | | **91.67%** | | **91.67%** | | **100.00%** | | **86.11%** | |
| **Domain 2:Stakeholder Involvement** | 11 | 12 | 14 | 14 | 10 | 11 | 17 | 13 | 17 | 15 | 21 | 13 | 12 | 13 | 14 | 11 | 19 | 15 |
| *The scaled domain score* | **47.22%** | | **61.11%** | | **41.67%** | | **66.67%** | | **72.22%** | | **77.78%** | | **52.78%** | | **52.78%** | | **77.78%** | |
| **Domain 3:Rigour of Development** | 22 | 36 | 31 | 48 | 18 | 17 | 25 | 19 | 54 | 56 | 41 | 39 | 14 | 11 | 31 | 22 | 21 | 15 |
| *The scaled domain score* | 43.75% | | 65.63% | | 19.79% | | 29.17% | | 97.92% | | 66.67% | | 9.38% | | 38.54% | | 20.83% | |
| **Domain 4:Clarity of Presentation** | 21 | 17 | 20 | 18 | 21 | 14 | 20 | 18 | 21 | 21 | 21 | 18 | 21 | 18 | 21 | 14 | 21 | 18 |
| *The scaled domain score* | **88.89%** | | **88.89%** | | **80.56%** | | **88.89%** | | **100%** | | **91.67%** | | **91.67%** | | **80.56%** | | **91.67%** | |
| **Domain 5:Applicability** | 6 | 13 | 10 | 23 | 8 | 16 | 18 | 14 | 19 | 28 | 19 | 18 | 12 | 14 | 13 | 16 | 14 | 14 |
| *The scaled domain score* | **22.92%** | | **52.08%** | | **33.33%** | | **50.00%** | | **81.25%** | | **60.41%** | | **37.5%** | | **43.75%** | | **41.67%** | |
| **Domain 6:Editorial Independence** | 11 | 14 | 11 | 14 | 12 | 14 | 10 | 14 | 13 | 14 | 12 | 14 | 9 | 14 | 11 | 14 | 9 | 14 |
| *The scaled domain score* | **87.5%** | | **87.5%** | | **91.67%** | | **87.5%** | | **95.83%** | | **91.67%** | | **79.17%** | | **87.50%** | | **79.17%** | |

A1: Appraiser 1; A2: Appraiser 2.
