# Supplementary material for: An evidence map of clinical practice guideline recommendations and quality of non-pharmaceutical interventions for post-stroke emotional disorders
Source: Front Neurol. 2025 Jun 9;16:1580799. doi: 10.3389/fneur.2025.1580799 (PMC12183077; doi:10.3389/fneur.2025.1580799)
Supplement: Supplementary file 3 [file Table_3.docx]

**S Table 3. Number of reporting items in CPGs by RIGHT checklist**

|  | **UWHMC,**  **2012** | **AHA/ASA,**  **2017** | **CSPM,**  **2018** | **CGHCMRA,**  **2019** | **CSC,**  **2020** | **CSA,**  **2020** | **HMHA,**  **2020** | **CBNIRC,**  **2022** | **BCPA,**  **2023** |
| --- | --- | --- | --- | --- | --- | --- | --- | --- | --- |
| **Domain 1: Basic information** | **5** | **6** | **6** | **6** | **6** | **6** | **6** | **6** | **6** |
|  | **83.33%** | **100.00%** | **100.00%** | **100.00%** | **100.00%** | **100.00%** | **100.00%** | **100.00%** | **100.00%** |
| 1a—Identifies the report as a guideline, that is, with “guideline(s)” or “recommendation(s)” in the title | No | Yes | Yes | Yes | Yes | Yes | Yes | Yes | Yes |
| 1b—Describes the year of publication of the guideline | Yes | Yes | Yes | Yes | Yes | Yes | Yes | Yes | Yes |
| 1c—Describes the focus of the guideline, such as screening, diagnosis, treatment, management, prevention or others | Yes | Yes | Yes | Yes | Yes | Yes | Yes | Yes | Yes |
| 2—Provides a summary of the recommendations contained in the guideline Abbreviations and acronyms | Yes | Yes | Yes | Yes | Yes | Yes | Yes | Yes | Yes |
| 3—Defines new or key terms, and provides a list of abbreviations and acronyms if applicable. | Yes | Yes | Yes | Yes | Yes | Yes | Yes | Yes | Yes |
| 4—Identifies at least one corresponding developer or author who can be contacted about the guideline | Yes | Yes | Yes | Yes | Yes | Yes | Yes | Yes | Yes |
| **Domain 2: Background** | **5** | **6** | **5** | **7** | **7** | **7** | **6** | **7** | **7** |
|  | **62.50%** | **75.00%** | **62.50%** | **87.50%** | **87.50%** | **87.50%** | **75.00%** | **87.50%** | **87.50%** |
| 5—Describes the basic epidemiology of the problem | Yes | Yes | Yes | Yes | Yes | Yes | Yes | Yes | Yes |
| 6—Describes the aim(s) of the guideline and specific objectives | Yes | Yes | Yes | Yes | Yes | Yes | Yes | Yes | Yes |
| 7a—Describes the primary population(s) that is addressed by the recommendation(s) in the guideline. | Yes | Yes | Yes | Yes | Yes | Yes | Yes | Yes | Yes |
| 7b—Describes any subgroups that are given special consideration in the guideline | No | No | No | No | No | No | No | Yes | No |
| 8a—Describes the intended primary users of the guideline and other potential users of the guideline | Yes | Yes | Yes | Yes | Yes | Yes | Yes | Yes | Yes |
| 8b—Describes the setting(s) for which the guideline is intended, such as primary care, low- and middle-income countries, or in-patient facilities | No | No | No | Yes | Yes | Yes | No | Yes | Yes |
| 9a—Describes how all contributors to the guideline development were selected and their roles and responsibilities | No | Yes | No | Yes | Yes | Yes | Yes | No | Yes |
| 9b—Lists all individuals involved in developing the guideline, including their title, role(s) and institutional affiliation(s). | Yes | Yes | Yes | Yes | Yes | Yes | Yes | Yes | Yes |
| **Domain 3: Evidence** | **2** | **1** | **1** | **0** | **4** | **4** | **0** | **1** | **0** |
|  | **40.00%** | **20.00%** | **20.00%** | **0.00%** | **80.00%** | **80.00%** | **0.00%** | **20.00%** | **0.00%** |
| 10a—States the key questions that were the basis for the recommendations in PICO (population, intervention, comparator, and outcome) or other formats as appropriate. | No | No | No | No | Yes | Yes | No | No | No |
| 10b—Indicates how the outcomes were selected and sorted | No | No | No | No | No | No | No | No | No |
| 11a—Indicates whether the guideline is based on new systematic reviews done specifically for this guideline or whether existing systematic reviews were used. | Yes | Yes | Yes | No | Yes | Yes | No | No | No |
| 11b—If the guideline developers used existing systematic reviews, references these and describes how those reviews were identified and assessed and whether they were updated. | No | No | No | No | Yes | Yes | No | No | No |
| 12—Describes the approach used to assess the certainty of the body of evidence. | Yes | No | No | No | Yes | Yes | No | Yes | No |
| **Domain 4:Recommendations** | **3** | **2** | **1** | **1** | **3** | **3** | **1** | **2** | **3** |
|  | **42.86%** | **28.57%** | **14.29%** | **14.29%** | **42.86%** | **42.86%** | **14.29%** | **28.57%** | **42.86%** |
| 13a—Provides clear, precise, and actionable recommendations | Yes | Yes | Yes | Yes | Yes | Yes | Yes | Yes | Yes |
| 13b—Presents separate recommendations for important subgroups | Yes | Yes | No | No | No | No | No | Yes | No |
| 13c—Indicates the strength of recommendations and the certainty of the supporting evidence. | No | No | No | No | Yes | Yes | No | No | No |
| 14a—Describes whether values and preferences of the target population(s) were considered in the formulation of each recommendation. | No | No | No | No | No | Yes | No | No | Yes |
| 14b—Describes whether cost and resource implications were considered in the formulation of recommendations. | Yes | No | No | No | No | No | No | No | No |
| 14c—Describes other factors taken into consideration when formulating the recommendations, such as equity, feasibility and acceptability. | No | No | No | No | No | No | No | No | No |
| 15—Describes the processes and approaches used by the guideline development group to make decisions | No | No | No | No | Yes | No | No | No | Yes |
| **Domain 5: Review and quality assurance** | **0** | **2** | **0** | **0** | **2** | **1** | **0** | **0** | **0** |
|  | **0.00%** | **100.00%** | **0.00%** | **0.00%** | **100.00%** | **50.00%** | **0.00%** | **0.00%** | **0.00%** |
| 16—Indicates whether the draft guideline underwent independent review and, if so, how this was executed and the comments considered and addressed | No | Yes | No | No | Yes | Yes | No | No | No |
| 17—Indicates whether the guideline was subjected to a quality assurance process. If yes, describe the process | No | Yes | No | No | Yes | No | No | No | No |
| **Domain 6: Funding, declaration and management of interest** | **1** | **2** | **2** | **1** | **4** | **2** | **0** | **3** | **0** |
|  | **25.00%** | **50.00%** | **50.00%** | **25.00%** | **100.00%** | **50.00%** | **0.00%** | **75.00%** | **0.00%** |
| 18a—Describes the specific sources of funding for all stages of guideline development. | No | No | No | No | Yes | Yes | No | Yes | No |
| 18b—Describes the role of funder(s) in the different stages of guideline development and in the dissemination and implementation of the recommendations. | No | No | No | No | Yes | No | No | Yes | No |
| 19a—Describes what types of conflicts (financial and non-financial) were relevant to guideline development | Yes | Yes | Yes | Yes | Yes | Yes | No | Yes | No |
| 19b—Describes how conflicts of interest were evaluated and managed and how users of the guideline can access the declarations. | No | Yes | Yes | No | Yes | No | No | No | No |
| **Domain 7: Other information** | **1** | **2** | **2** | **0** | **2** | **2** | **1** | **3** | **3** |
|  | **33.33%** | **66.67%** | **66.67%** | **0.00%** | **66.67%** | **66.67%** | **33.33%** | **100.00%** | **100.00%** |
| 20—Describes where the guideline, its appendices, and other related documents can be accessed. | No | No | No | No | Yes | Yes | No | Yes | Yes |
| 21—Describes the gaps in the evidence and/or provides suggestions for future research. | Yes | Yes | Yes | No | Yes | Yes | Yes | Yes | Yes |
| 22—Describes any limitations in the guideline development process, and indicates how these limitations might have affected the validity of the recommendations. | No | Yes | Yes | No | No | No | No | Yes | Yes |
| **Number of reported items** | **17** | **21** | **17** | **15** | **28** | **25** | **14** | **22** | **19** |
|  | **48.57****%** | **60.00%** | **48.57%** | **42.86%** | **80.00%** | **71.43%** | **40.00%** | **62.86%** | **54.29%** |
