# Supplementary material for: An evidence map of clinical practice guideline recommendations and quality of non-pharmaceutical interventions for post-stroke emotional disorders
Source: Front Neurol. 2025 Jun 9;16:1580799. doi: 10.3389/fneur.2025.1580799 (PMC12183077; doi:10.3389/fneur.2025.1580799)
Supplement: Supplementary file 4 [file Table_4.docx]

S Table 4. Details of grading systems

| **Guideline** | **Grading system** | **Level of evidence** | **Definition** |
| --- | --- | --- | --- |
| UWHMC | NR | Class I | Prospective, randomized, controlled clinical trial with masked outcome assessment, in a representative population. The following are required: (1) primary outcome clearly specified; (2) exclusion/inclusion criteria clearly defined; (3c adequate accounting for drop-outs and cross-overs with low numbers to minimize bias; (4) relevant baseline characteristics are presented and substantially equivalent. |
|  |  | Class II | Prospective matched group cohort study in a representative population with masked outcome assessment that meets (1) to (2) above OR a randomized controlled trial that lacks one of the criteria. |
|  |  | Class III | All other controlled trials (including well-defined natural history controls or patients serving their own controls) in a representative population, where outcome is independently assessed or independently derived by objective outcome measurement. |
|  |  | Class IV | Evidence from uncontrolled studies, case series, case reports or expert opinion. |
| CSC | ACCP | A | Evidence from a meta-analysis of randomized controlled trials or consistent findings from two or more randomized controlled trials. Desirable effects clearly outweigh undesirable effects or undesirable effects clearly outweigh desirable effects. |
|  |  | B | Evidence from a single randomized controlled trial or consistent findings from two or more well-designed non-randomized and/or non-controlled trials, and large observational studies. Desirable effects outweigh or are closely balanced with undesirable effects or undesirable effects outweigh or are closely balanced with desirable effects. |
|  |  | C | Writing group consensus and/or supported by limited research evidence. Desirable effects outweigh or are closely balanced with undesirable effects or undesirable effects outweigh or are closely balanced with desirable effects as determined by writing group consensus. Recommendations assigned a Level-C evidence may be key system drivers supporting other recommendations, and some may be expert opinion based on common, new or emerging evidence or practice patterns. |
|  |  | Clinical Consideration | Reasonable practical advice provided by consensus of the writing group on specific clinical issues that are common and/or controversial and lack research evidence to guide practice. |
| CSA | GRADE | A | We are very confident that the true effect lies close to that of the estimate of the effect. |
|  |  | B | We are moderately confident in the effect estimate: The true effect is likely to be close to the estimate of the effect, but there is a possibility that it is substantially different. |
|  |  | C | We are moderately confident in the effect estimate: The true effect is likely to be close to the estimate of the effect, but there is a possibility that it is substantially different. |
|  |  | D | We have very little confidence in the effect estimate: The true effect is likely to be substantially different from the estimate of effect. |
| CBNIRC | NR | Ia | Evidence from meta-analysis of randomized controlled trials (RCTs). |
|  |  | Ib | Evidence from at least one RCT. |
|  |  | IIa | Evidence from at least one controlled study without randomization. |
|  |  | IIb | Evidence from at least one quasi-experimental study. |
|  |  | III | Evidence from nonexperimental descriptive studies, such as correlation studies comparative studies, and case -control studies. |
|  |  | IV | Evidence from opinions and/or clinical experience of respected authorities or expert committee reports. |
|  | **Grading system** | **Strength of evidence** | **Definition** |
| CSA | GRADE | Ⅰ | A strong recommendation is one for which guideline panel is confident that the desirable effects of an intervention outweigh its undesirable effects (strong recommendation for an intervention) or that the undesirable effects of an intervention outweigh its desirable effects (strong recommendation against an intervention). |
|  |  | Ⅱ | A weak recommendation is one for which the desirable effects probably outweigh the undesirable effects (weak recommendation for an intervention) or undesirable effects probably outweigh the desirable effects (weak recommendation against an intervention) but appreciable uncertainty exists. |
| CBNIRC | NR | A | Directly based on Category I evidence. |
|  |  | B | Directly based on Category II evidence or extrapolated recommendation from Category I evidence. |
|  |  | C | Directly based on Category III evidence or extrapolated recommendation from category I or II evidence. |
|  |  | D | Directly based on Category IV evidence or extrapolated recommendation from Category I, II or III evidence. |
|  |  | S | Standard of care. |

ACCP: American College of Chest Physicians; GRADE: Grading of Recommendations Assessment, Development and Evaluation; NR: The grading system name Not Reported.
