# Supplementary material for: An evidence map of clinical practice guideline recommendations and quality of non-pharmaceutical interventions for post-stroke emotional disorders
Source: Front Neurol. 2025 Jun 9;16:1580799. doi: 10.3389/fneur.2025.1580799 (PMC12183077; doi:10.3389/fneur.2025.1580799)
Supplement: Supplementary file 5 [file Table_5.docx]

**S Table 5. Evidence quality and recommendation strength**

| Recommended therapies | UWHMC | | AHA/ASA | | CSPM | | CGHCMRA | | CSC | | CSA | | HMHA | | CBNIRC | | BCPA | |
| --- | --- | --- | --- | --- | --- | --- | --- | --- | --- | --- | --- | --- | --- | --- | --- | --- | --- | --- |
|  | LoE | SoR | LoE | SoR | LoE | SoR | LoE | SoR | LoE | SoR | LoE | SoR | LoE | SoR | LoE | SoR | LoE | SoR |
| CBT | Ⅱ | / | / | / | / | / | / | / | B | / | / | / | / | / | Ⅰa | B | / | / |
| BPI | / | / | / | / | / | / | / | / |  | / | / | / | / | / | / | / | / | / |
| SSI | / | / | / | / | / | / | / | / | / | / | B | IIb(supplementary therapy) | / | / | / | / | / | / |
| BPT | / | / | / | / | / | / | / | / | A(Combined drugs) | / | / | / | / | / | / | / | / | / |
| MT | / | / | / | / | / | / | / | / | / | / | B | II(Combined drugs) | / | / | / | / | / | / |
| NTTCM | / | / | / | / | / | / | / | / | / | / | / | / | / | / | Ⅲ | C | / | / |
| MBSR | / | / | / | / | / | / | / | / | / | / | A | I | / | / | / | / | / | / |
| rTMS | / | / | / | / | / | / | / | / | / | / | B | IIa(Combined drugs) | / | / | Ⅱa | B | / | / |
| HBOT | / | / | / | / | / | / | / | / | / | / | B | IIa(Combined drugs) | / | / | / | / | / | / |

**CBT**: Cognitive behavioral interventions; **BPI**: Brief psychosocial interventions; **BPT**: Balancing Psychotherapy; **MT**: Music therapy; **SSI**: Social support interventions; **NTTC**M: Non-pharmacological therapies of Traditional Chinese Medicine; **MBSR**: Mindfulness-Based Stress Reduction; **rTMS**: repetitive transcranial magnetic stimulation; **HBOT**: Hyperbaric oxygen therapy; **LoE**: Level of evidence; **SoR**: Strength of recommendation; **“/”**:Not reported; The strength of recommendation is represented by green, and the darker the color is, the stronger the recommendation; The level of evidence is represented by blue, and the darker the color is, the higher the level of evidence.
